# Effects of cannabidiol on brain excitation and inhibition systems; a randomised placebo-controlled single dose trial during magnetic resonance spectroscopy in adults with and without autism spectrum disorder

**DOI:** 10.1038/s41386-019-0333-8

**Published:** 2019-02-06

**Authors:** Charlotte Marie Pretzsch, Jan Freyberg, Bogdan Voinescu, David Lythgoe, Jamie Horder, Maria Andreina Mendez, Robert Wichers, Laura Ajram, Glynis Ivin, Martin Heasman, Richard A. E. Edden, Steven Williams, Declan G. M. Murphy, Eileen Daly, Gráinne M. McAlonan

**Affiliations:** 1 Department of Forensic and Neurodevelopmental Sciences, Institute of Psychiatry, Psychology & Neuroscience, King’s College London, London, UK; 2 Department of Neuroimaging Sciences, Institute of Psychiatry, Psychology & Neuroscience, King’s College London, London, UK; 3 South London and Maudsley NHS Foundation Trust Pharmacy, London, UK; 4 Russel H Morgan Department of Radiology and Radiological Science, Johns Hopkins Medical Institutions, Baltimore, MD USA

**Keywords:** Development of the nervous system, Drug development, Autism spectrum disorders

## Abstract

There is increasing interest in the use of cannabis and its major non-intoxicating component cannabidiol (CBD) as a treatment for mental health and neurodevelopmental disorders, such as autism spectrum disorder (ASD). However, before launching large-scale clinical trials, a better understanding of the effects of CBD on brain would be desirable. Preclinical evidence suggests that one aspect of the polypharmacy of CBD is that it modulates brain excitatory glutamate and inhibitory γ-aminobutyric acid (GABA) levels, including in brain regions linked to ASD, such as the basal ganglia (BG) and the dorsomedial prefrontal cortex (DMPFC). However, differences in glutamate and GABA pathways in ASD mean that the response to CBD in people with and without ASD may be not be the same. To test whether CBD ‘shifts’ glutamate and GABA levels; and to examine potential differences in this response in ASD, we used magnetic resonance spectroscopy (MRS) to measure glutamate (Glx = glutamate + glutamine) and GABA+ (GABA + macromolecules) levels in 34 healthy men (17 neurotypicals, 17 ASD). Data acquisition commenced 2 h (peak plasma levels) after a single oral dose of 600 mg CBD or placebo. Test sessions were at least 13 days apart. Across groups, CBD increased subcortical, but decreased cortical, Glx. Across regions, CBD increased GABA+ in controls, but decreased GABA+ in ASD; the group difference in change in GABA + in the DMPFC was significant. Thus, CBD modulates glutamate-GABA systems, but prefrontal-GABA systems respond differently in ASD. Our results do not speak to the efficacy of CBD. Future studies should examine the effects of chronic administration on brain and behaviour, and whether acute brain changes predict longer-term response.

## Introduction

Autism spectrum disorder (ASD) affects up to 1 in 59 individuals [1]. Of those affected, 70% also have co-occurring conditions such as epilepsy [2], and mood and anxiety disorders [3]. This incurs a high cost to the individual and society: on average the lifespan of individuals with ASD is reduced by 20 years [4]. Given the lack of effective pharmacological treatments, researchers have therefore begun to explore alternative options. These include cannabis and its major non-intoxicating component cannabidiol (CBD), which is derived from the *cannabis sativa* plant [5].

CBD has already been trialled in several disorders. For instance, preliminary evidence suggests that CBD may improve spasticity [6], pain, sleep disturbances [7], and mobility [8] in multiple sclerosis (MS); and alleviate anxiety symptoms in social phobia [9]. Moreover, alongside anecdotal accounts and case series reports of benefits from medical marijuana in ASD [10], there is evidence that CBD: (i) reduces seizure frequency in two epilepsy syndromes associated with autistic symptoms: Dravet Syndrome and Lennox–Gastaut syndrome [11–13]; and (ii) improves ASD-like social deficits in a mouse model of Dravet Syndrome [14]. This suggests that CBD may be worth further investigation in idiopathic ASD. However, before embarking on large-scale clinical trials, a better understanding of how CBD acts on the human brain, and especially in ASD, would be desirable.

CBD has multiple targets, but one aspect of its polypharmacy may be to help regulate excitatory glutamate (E) and inhibitory γ-aminobutyric acid (GABA) (I) transmission, which may influence the activity of excitatory and inhibitory signalling pathways: For example, CBD facilitates glutamate and GABA neurotransmission across the brain through agonism at the transient receptor potential vanilloid type 1 (TRPV1) receptor [15, 16]. Moreover, CBD may increase GABAergic transmission by antagonism at the G protein-coupled receptor 55 (GPR55), and especially in the basal ganglia [14] (BG). In contrast, CBD is thought to be an agonist at prefrontal 5-HT1A receptors, where it suppresses glutamate and GABA transmission [17, 18]. In sum, CBD may act on targets throughout the brain, but especially in the BG and the prefrontal cortex. These actions of CBD upon glutamate-GABA pathways may be especially important in ASD, where post mortem, genetic, and in vivo proton magnetic resonance spectroscopy (MRS) studies have shown abnormalities in both prefrontal and BG glutamate and GABA pathways [19–21]; both regions have also been repeatedly linked to ASD core symptoms [21, 22]. Thus, CBD could well impact on prefrontal and BG Glx and GABA levels in ASD, but not necessarily in the same manner as in unaffected individuals (with intact glutamate-GABA systems). However, no-one has investigated this directly.

Therefore, in this study, we tested the hypotheses that CBD impacts on human in vivo glutamate and GABA levels in the BG and dorsomedial prefrontal cortex (DMPFC); but that the response is atypical in ASD. To achieve this, we compared MRS measures of glutamate and GABA in men with and without ASD following a single oral dose of 600 mg CBD or a matched placebo (at least 2 weeks apart) in a randomised double-blind, cross-over design.

## Materials and methods

### Procedure

This research was conducted in accordance with the Declaration of Helsinki, at the Institute of Psychiatry, Psychology, and Neuroscience (IoPPN) at De Crespigny Park, SE5 8AF, London, UK (August 2016 to August 2018). The Medicines and Health Research Authority (MHRA) in the UK confirmed the study design was not a Clinical Trial and ethical approval for this study was provided by the King’s College London Research Ethics Committee, study reference HR15/162744. All participants provided written informed consent. Every participant took part in all aspects of this case-control study.

This placebo-controlled, randomised, double-blind, repeated-measures, cross-over case-control study was conducted as part of a larger investigation into the role of phytocannabinoids in ASD; clinicaltrials.gov (identifier: NCT03537950, entry name: HR15-162744). Placebo (PLC) or CBD was allocated in a pseudo-randomised order, so that approximately half in each group attended a placebo visit before CBD; and half attended a CBD visit before placebo. The randomisation was implemented by Prof McAlonan using https://www.random.org/. Participants and researchers directing the study were blind to the assignment. Participants attended for two visits. To allow for drug wash-out, visits were separated each by a minimum of 13 days, with all attempts made to keep between-visit time consistent across all visits and participants. Moreover, the acquisition of data from both groups was mostly overlapping during the same period. On each visit, urine samples were taken to screen for illicit substances (a full list is included below). Subsequently, participants underwent a brief health check, received a liquid oral dose of the pharmacological probe (600 mg of CBD; in line with previous single dose studies of CBD adults (e.g. [23]) or a matched placebo, both provided by GW Research Ltd, Cambridge, UK), and a second brief health check to test for potential acute adverse reactions/side effects. Participants underwent scanning timed to coincide with peak plasma (2 h) concentration. After the scan, participants received a third health check to ensure they had experienced no ill-effects and were fit to leave the department.

### Participants

Potential participants were excluded if they had a comorbid major psychiatric or medical disorder affecting brain development (e.g. schizophrenia or epilepsy), a history of head/brain injury, a genetic disorder associated with ASD (e.g. tuberous sclerosis or Fragile X syndrome), or an IQ below 70. We also excluded participants who were reliant on receiving regular medication known to directly modulate glutamate and GABA systems. However, we included participants on other medications which are commonly prescribed in ASD: one person with ASD who took a single dose of Ritalin on the morning of each study visit, and one person with ASD who took a single dose of sertraline on the morning of each study visit. We asked participants to abstain from using cannabis and/or other illicit substances in the month prior to scanning, and from drinking alcohol on the day prior to testing. We also carried out Urine Drug Screening on each test day. Data from individuals who screened positive for these substances were excluded. Thus, we initially retained data from 34 subjects (neurotypical control *n* = 17, ASD *n* = 17) (see Table 1 for demographics); this sample size was sufficient to detect a 10% E-I shift (where ‘shift’ means a change in a component of the Glx-GABA metabolite pool) at a power of 0.8 and a significance level of *α* = 0.05, based on a power analysis using previous findings in the department [19]. All participants had an IQ over 70. All participants in the ASD group had a clinical diagnosis of ASD made according to ICD10 research criteria [24–26], and severity of symptoms was confirmed using standardised research diagnostic instruments (Autism Diagnostic Observation Schedule, ADOS; and Autism Diagnostic Interview-Revised, ADI-R).

**Table 1:** Participant demographics for all subjects are reported including standard deviations (except for N)

Demographic measure	TD	ASD	*F*(dof)	*p*-value
*N* (M/F)	17 (17/0)	17 (17/0)		
Age in years	28.47 (6.55)	31.29 (9.94)	*F*(1) = 0.956	*p* = 0.335
Days between visits	34.82 (24.99)	36.44 (20.53)	*F*(1) = 0.041	*p* = 0.841
FSIQ	124.59 (12.7)	111.35 (18.80)	*F*(1) = 5.781	***p*** = 0.022

Significant between-group differences are highlighted in bold

*ASD* autism spectrum disorder, *F(dof)* F statistic and degrees of freedom, *F* female, *FSIQ* full scale intelligence quotient, *M* male, *N*, participant number, *TD* typically developing individuals

### Imaging data acquisition

All imaging data were acquired on a 3T GE Excite II magnetic resonance imaging (MRI) scanner (GE Medical Systems, Milwaukee, WI, USA). The scanning protocol included a structural MRI scan acquired using a 3D inversion recovery prepared fast spoiled gradient recalled (IR-FSPGR) sequence (slice thickness = 1.1 mm, spatial positions = 124, flip angle = 20°, field of view (FoV) = 280 mm, echo time (TE) = 2.844 ms, repetition time (TR) = 7.068 ms, inversion time = 450 ms, matrix = 256 × 256). This structural scan was conducted to obtain information used during the preprocessing of the spectroscopy scan. The scanning protocol further included a spectroscopy scan based on the MEshcher-GArwood Point RESolved Spectroscopy (MEGA-PRESS) sequence [27]. We acquired data (44 averages) from two voxels: the first was positioned in the BG (echo time (TE) = 68 ms, repetition time (TR) = 1800 ms, voxel size = 35*30*25 mm^3^).

This voxel was placed with the anterior border initially abutting the anterior portion of the left lentiform nucleus, and as medial as possible, to avoid the ventricles as much as possible. Thus, taking into account slight inter-individual anatomical differences, this voxel was on average composed as follows: the white matter (WM), primarily included the internal capsule and part of the corpus callosum. The grey matter (GM), included the BG [~55%], the thalamus [~25%] and the insula [~20%].

The second voxel was positioned in the DMPFC (TE = 68 ms, TR = 2000 ms, voxel size = 25*40*30 mm^3^). This voxel was placed in the midline, avoiding the corpus callosum. Resultantly, given inter-individual variance, this voxel was composed as follows: the WM included the corpus callosum and cingulum; while the GM included the anterior part of the cingulate gyrus.

**Fig. 1:**
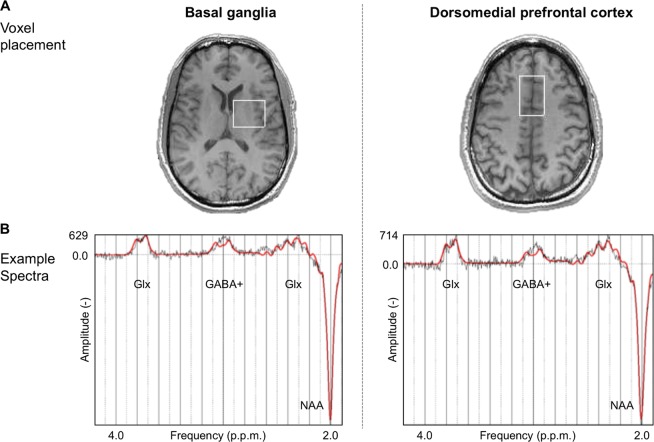
Magnetic resonance spectroscopy (MRS) representative voxel placement and example spectra. **a** MRS voxel of interest (outlined in white) in the basal ganglia and the dorsomedial prefrontal cortex. **b** Example spectroscopy spectra from each voxel. Glx glutamate + glutamine, GABA + γ-aminobutyric acid + macromolecules, NAA N-acetyl-aspartate, p.p.m parts per million

Representative voxel positions are shown in Fig. 1.

### Urine test

To evaluate presence or absence of illicit substances that could confound potential effects of the pharmacological probes tested in this study, we performed liquid chromatography-mass spectrometry (LC-MS) analysis on urine samples provided by each participant before the drug administration. Participants that showed positive results for any of the drugs tested, including Amphetamines (Amphetamine, Methamphetamine, MDMA/Ecstasy), Benzodiazepines, Cannabis, Cocaine (as benzoylecgonine), Methadone and its metabolite EDDP, and Opioids (6-Monoacetylmorphine, Morphine, Codeine, Dihydrocodeine), were excluded from the analysis, resulting in the exclusion of four subjects (two controls, two ASD) from the original sample.

### Data processing

#### Structural data processing

T1-weighted structural MRI volumes were inspected manually to ensure adequate signal-to-noise ratio (SNR) and absence of motion artefacts. Subsequently, structural volumes were normalised to Montreal Neurological Institute (MNI) space, and segmented into GM, WM, and CSF, to obtain percentage measures of tissue composition in each individual MRS voxel, using positional coordinates embedded in the raw spectra data files.

#### MRS data processing

MRS data were pre-processed using in-house scripts adapted from FID-A [28], which prepared the data for reading into the main processing software. This included conversion of data to the required file format, combination of receiver channels, removal of ‘bad’ averages (>4 standard deviations), frequency drift correction (alignment of averages), separation and visualisation of the edit on/off spectra, and their subtraction to generate the difference spectrum. Each spectrum was manually inspected to ensure adequate SNR, as well as the absence of artefacts [26, 28]. Representative example spectra are displayed in Fig. 1.

MRS data were then processed using LCModel v6.3-1 L software (Stephen Provencher Incorporated, Oakville, Canada). LCModel uses a linear combination of model spectra derived from metabolite solutions in vitro to analyse the major resonances of in vivo spectra. For this analysis, we used a basis set (mega-press-3T-1) to determine the concentrations of GABA+ (which comprises GABA plus macromolecules), glutamine, glutamate, glutathione (GSH), N-acetyl-aspartate (NAA), N-acetyl-aspartylglutamate (NAAG), NAA + NAAG, Glx (Glu + Gln), and GSH + Glu + Gln in each voxel; however, for this analysis, we focused solely on GABA+ and Glx.

In MRS, partial volume effects (different proportions of GM, WM, and CSF in the MRS voxels) are a potential confound, especially given previously reported volumetric differences between autistic and neurotypical individuals [29]. To account for partial volume effects, we therefore corrected all metabolites for GM, WM, and CSF percentages. Assuming that CSF only contains negligible quantities of the metabolites of interest, the calculations were as follows: LCModel assumes a voxel is 100% WM with a water concentration (WCONC) of 35880 mM and corrects each metabolite value (where F stands for fraction) using the factor: (43300*F_GM_ + 35880*F_WM_ + 55556*F_CSF_)/(1-F_CSF_). To correct for the value of water concentration being used in the processing through LCModel, we divided values by an individual correction factor (35880), arriving at (1.207*F_GM_ + F_WM_ + 1.548*F_CSF_)/(1-F_CSF_). Therefore, in summary, the corrected metabolite values were obtained by multiplying the raw metabolite values by this correction. Since we did not measure relaxation times for tissue water and metabolites, these were not corrected for—with the exception of assuming the tissue water relaxation time (*T*_2_ = 80 ms) [30].

To further ensure the robustness of our findings, we excluded all measurements of GABA+ and Glx (Glx = glutamate + glutamine) where the LCModel Cramér-Rao lower bound (CRLB) estimates exceeded 15% from further analysis (LCModel manual, Stephen Provencher Incorporated, Oakville, Canada). This resulted in the exclusion of a total of eleven data points from six ASD participants from the original sample. The spread across voxels and conditions (placebo/CBD) was as follows: 1: BG Glx_PLC_; 2: DMPFC GABA + _PLC_ & Glx_PLC_; 3: DMPFC GABA + _CBD_ & Glx_CBD_; 4: DMPFC GABA + _CBD_ & Glx_CBD_; 5 (also excluded due to positive drug screening): DMPFC GABA + _PLC_ & Glx_CBD_; 6: DMPFC Glx_CBD_ & GABA + _CBD_.

### Statistical analysis

Demographic measures (age, IQ) and baseline levels of Glx and GABA+ in each region of interest were compared using a one-way ANOVA (significance level *p* < 0.05).

To test the primary hypothesis that CBD impacts on E-I balance in our two brain regions of interest (BG and DMPFC), differences in mean metabolite concentrations were calculated using two 2 × 2 × 2 mixed-model ANOVAs with group (neurotypicals, ASD) as between-subject factor, voxel (BG, DMPFC) and drug (PLC, CBD) as within-subject factors, and the respective metabolite (Glx, GABA+) as the dependent variable. Our planned comparisons tested a priori predictions that CBD would impact upon Glx and GABA+; and that there would be differences in the response of participants with and without ASD. With the caveat that Bonferroni testing can be overly conservative, for completeness however, we also report a Bonferroni corrected *p*-value alongside any significant (uncorrected) results.

However, this repeated-measures approach is impacted by missing data (one missing/poor quality data point from either voxel during either placebo or drug condition results in data from that individual being omitted); also there is a possibility that our results were influenced by the different T1-weighting in the cortical and subcortical voxels. Therefore, following this overall analysis we conducted secondary post hoc two-by-two mixed-model ANOVAs with group (neurotypicals, ASD) as a between-subject factor, and drug (PLC, CBD) as a within-subject factor for each metabolite in each region separately and examined any group difference in the *change* in each using as much of the available data as possible. Thus, for Glx measures in both BG and DMPFC, ASD *n* = 13, neurotypicals *n* = 17; for GABA+ measures in the DMPFC, ASD *n* = 11, neurotypicals *n* = 17; and for GABA+ measures in BG, ASD *n* = 16, neurotypicals *n* = 17.

All analyses were performed using SPSS 24.00 software (SPSS, Chicago, IL, USA). Graphs displaying results were produced using GraphPad Prism version 7 for Mac, GraphPad Software, La Jolla, CA, USA, www.graphpad.com.

## Results

### Demographics

Groups did not differ significantly in age (*F*(1) = 0.956, *p* = 0.335); but, as is commonly reported, individuals with ASD had a slightly lower IQ than neurotypical controls, and this difference was significant (*F*(1) = 5.781, *p* = 0.022) (as summarised in Table 1). Therefore, to be sure that our findings were not influenced by IQ, we investigated the relationship between drug-induced shifts (CBD-PLC) in metabolite levels (Glx and GABA+) and IQ. As expected, there were no significant correlations across the whole group (*r* < 0.095, *p* > 0.350), in ASD alone (*r* < −0.008, *p* > 0.698) nor in the neurotypicals alone (*r* < 0.068, *p* > 0.235), suggesting that the difference in IQ did not influence the results. No participant experienced any subjective or objective ill-effects/harm following administration of the study drug.

### Tissue composition and data quality

Tissue percentage (not excluding omitted spectra) differed between groups for BG PLC GM (*F*(1) = 7.307, *p* = 0.011) and for BG PLC WM (*F*(1) = 9.345, *p* = 0.004), but not for other tissues or drug conditions (as summarised in Table 2). This is unsurprising, as previous studies have suggested morphological differences in the BG in autistic compared to neurotypical individuals [31]. In our statistical analysis we corrected all metabolite values accordingly.

**Table 2:** Absolute values (and standard deviations) for percentages of grey and white matter and cerebrospinal fluid in the voxels of interest

Voxel	Drug	Tissue	TD	ASD	*F*(dof)	*p*-value
BG	PLC	GM	42.53% (3.03%)	45.43% (3.21%)	*F*(1) = 7.307	***p*** = 0.011
		WM	50.47% (3.39%)	46.54% (4.08%)	*F*(1) = 9.345	***p*** = 0.004
		CSF	6.92% (1.33%)	7.95% (1.95%)	*F*(1) = 3.222	*p* = 0.082
	CBD	GM	43.24% (3.54%)	45.07% (3.64%)	*F*(1) = 2.157	*p* = 0.152
		WM	49.20% (4.66%)	47.29% (4.32%)	*F*(1) = 1.482	*p* = 0.233
		CSF	7.46% (2.16%)	7.56% (1.93%)	*F*(1) = 0.19	*p* = 0.890
DMPFC	PLC	GM	52.93% (2.21%)	52.38% (3.49%)	*F*(1) = 0.299	*p* = 0.589
		WM	27.24% (3.36%)	28.11% (3.65%)	*F*(1) = 0.527	*p* = 0.473
		CSF	19.73% (4.05%)	19.41% (2.57%)	*F*(1) = 0.076	*p* = 0.784
	CBD	GM	52.69% (2.86%)	52.84% (3.69%)	*F*(1) = 0.017	*p* = 0.898
		WM	27.32% (3.54%)	27.41% (4.14%)	*F*(1) = 0.004	*p* = 0.947
		CSF	19.89% (3.72%)	19.63% (2.53%)	*F*(1) = 0.048	*p* = 0.827

Significant between-group differences are highlighted in bold

*ASD* autism spectrum disorder, *BG* basal ganglia, *CBD* cannabidiol, *CSF* cerebrospinal fluid, *DMPFC* dorsomedial prefrontal cortex, *F(dof)* F statistic and degrees of freedom, *GM* grey matter, *PLC* placebo, *TD* typically developing individuals, *WM* white matter

To ensure that the [H]MRS data quality was equal between groups, we compared CRLB estimates for each metabolite (Glx, GABA+) in each voxel (excluding omitted spectra), using a one-way ANOVA. As expected, we found no significant differences (all *F*(1) ≤ 4.102, all *p* ≥ 0.052) (as summarised in Table 3).

**Table 3:** Absolute values (and standard deviations) for Cramér-Rao Lower Bound estimates for each metabolite in each voxel

Voxel	Drug	Metabolite	TD	ASD	*N* (TD, ASD)	*F*(dof)	*p*-value
BG	PLC	GABA+	4.56 (0.66)	4.82 (0.81)	17, 17	*F*(1) = 1.095	*p* = 0.303
		Glx	7.50 (2.09)	7.56 (2.25)	17, 16	*F*(1) = 0.007	*p* = 0.935
	CBD	GABA+	4.59 (0.62)	4.25 (1.43)	17, 16	*F*(1) = 0.782	*p* = 0.383
		Glx	7.82 (3.00)	6.75 (2.48)	17, 16	*F*(1) = 1.236	*p* = 0.275
DMPFC	PLC	GABA+	6.47 (0.87)	7.38 (2.06)	17, 16	*F*(1) = 2.751	*p* = 0.107
		Glx	5.68 (0.73)	5.88 (1.02)	17, 16	*F*(1) = 0.416	*p* = 0.524
	CBD	GABA+	6.29 (0.85)	7.69 (2.69)	17, 13	*F*(1) = 4.102	*p* = 0.052
		Glx	6.24 (1.95)	6.31 (1.55)	17, 13	*F*(1) = 0.12	*p* = 0.913

*ASD* autism spectrum disorder, *BG* basal ganglia, *CBD* cannabidiol, *DMPFC* dorsomedial prefrontal cortex, *F(dof)* F statistic and degrees of freedom, *GABA +*  g-aminobutyric acid + macromolecule, *Glx* glutamate + glutamine, *N* number of individuals, *PLC* placebo, *TD* typically developing individuals

In extended MRS studies, there is often a risk of ‘drift’, where the metabolite estimates on the same scanner change over long periods of time. For this reason, we compared the duration between scans (days between PLC and CBD scan) across the two groups. There was no significant difference in duration between visits (*F*(1) = 0.041, *p* = 0.841) in controls (34.82 ± 24.99) and ASD (36.44 ± 20.53) (see Table 1). Furthermore, scan date for each drug condition (PLC, CBD) was not correlated with the value of any metabolite at that drug condition (all Pearson’s *r* ≤ 0.299, all *p* ≥ 0.115), confirming that data acquisition was stable over time.

### Metabolite differences

#### Glx (glutamate+glutamine)

**Fig. 2:**
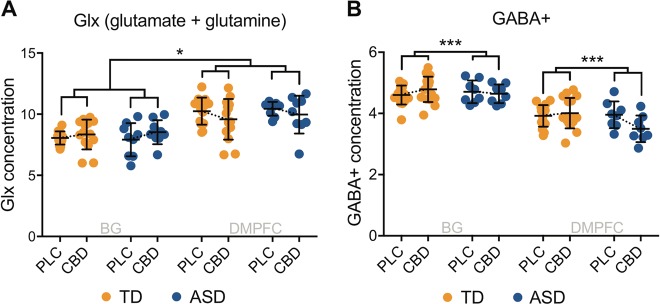
Glx (glutamate + glutamine) (14 neurotypicals, 9 autistic individuals) (**a**) and GABA+ (γ-aminobutyric acid + macromolecules) (16 neurotypicals, 8 autistic individuals) (**b**) in the basal ganglia and the dorsomedial prefrontal cortex for both groups in both drug conditions. Glx (**a**) and GABA+ (**b**) concentration represents the ratio of the Glx and GABA+ metabolite resonance area to the unsuppressed water resonance area, respectively. Dotted lines connect group means, which are indicated by black horizontal bars. Error bars represent standard deviations. ASD autism spectrum disorder, BG basal ganglia, CBD cannabidiol, DMPFC dorsomedial prefrontal cortex, PLC placebo, TD typically developed controls; * indicates a significance level at *p* ≤ 0.05; *** indicates a significance level at *p* ≤ 0.001

There were no significant between-group differences in baseline Glx in the BG (*F*(1) = 0.000, *p* = 0.993, *n* = 29) or in the DMPFC (*F*(1) = 0.196, *p* = 0.661, *n* = 32). There was however a significant voxel × drug interaction effect (*F*(1,21) = 5.235, *p*_uncorr_ = 0.033, partial eta squared (*η*^2^) = 0.200): in both groups, CBD increased Glx in the BG and decreased Glx in the DMPFC (as depicted in Fig. 2). This effect did not survive stringent Bonferroni-correction. Nonetheless, *p*_corr_ = 0.126 indicates at least an 87% likelihood that the observed effect was real.

Results of post hoc testing within each voxel separately were consistent with these findings, albeit at trend level. In the BG, CBD increased Glx in both groups (*F*(1,24) = 3.593, *p*_uncorr_ = 0.070, *η*^2^ = 0.130); in the DMPFC, CBD decreased Glx in both groups (*F*(1,26) = 4.030, *p*_uncorr_ = 0.055, *η*^2^ = 0.134). Thus, differences in the acquisition parameters for each region were unlikely to explain the overall results. Moreover, post-hoc within-subject comparisons of Glx changes (CBD-PLC) showed that there was no group-difference in Glx responsivity to CBD in the BG (*F*(1) = 0.602, *p*_uncorr_ = 0.445) nor in the DMPFC (*F*(1) = 0.006, *p*_uncorr_ = 0.937), confirming that Glx in adults with and without ASD responded to CBD in the same way.

#### GABA+

There were no significant between-group differences in baseline GABA+ in the BG (*F*(1) = 0.000, *p*_uncorr_ = 0.987, *n* = 33) or in the DMPFC (*F*(1) = 0.408, *p*_uncorr_ = 0.528, *n* = 30). There was however a significant group × drug interaction in both brain regions (*F*(1,22) = 13.506, *p*_uncorr_ = 0.001, *η*^2^ = 0.380). CBD increased GABA+ in the control group and decreased GABA+ in autistic individuals. This effect survived Bonferroni-correction (*p*_corr_ = 0.004). These findings are displayed in Fig. 2.

Post hoc testing in each voxel separately indicated that this result was largely driven by changes in the DMPFC, where there was a significant group × drug interaction effect (*F*(1,23) = 4.864, *p*_uncorr_ = 0.038, *η*^2^ = 0.175); and the group difference in CBD-induced change in GABA+ was significant in the DMPFC (*F*(1) = 6.510, *p*_uncorr_ = 0.017), but not in the BG.

Post hoc within-subject analyses of GABA+ changes (CBD-PLC) also confirmed a significant group difference in the DMPFC (*F*1) = 4.864, *p*_uncorr_ = 0.038), and but not the BG. This effect did not survive stringent Bonferroni-correction (*p*_corr_ = 0.14), but there was at least a 86% chance (*p*_corr_ = 0.14) it was real.

Finally, given that we excluded ASD participants, but not neurotypicals, on the basis of low CRLB estimates, we also reran our analysis including CRLB measures as a covariate and this did not materially alter the findings. Thus, GABA+ in adults with and without ASD responded to CBD in opposite directions, and especially in the cortex.

We note that secondary analyses confirmed that CBD did not alter the levels of other metabolites within the spectrum; namely we observed no significant group and drug main effects, and no group × drug interaction effects for GSH, NAA, NAAG, NAA + NAAG, and GSH + Glx.

## Discussion

Here we report that acute (single dose) CBD ‘shifts’ levels of the brain’s primary excitatory and inhibitory neurotransmitters in adults with and without ASD. In both groups, CBD increased Glx in the BG voxel and decreased it in the DMPFC voxel. In contrast, CBD had opposite effects on GABA+ in each group. Specifically, both in prefrontal and subcortical regions, CBD increased GABA+ in the controls but decreased GABA+ in ASD. Moreover, in line with some [19, 21], but not all previous MRS studies of glutamate and GABA in ASD [21, 32] in the BG and DMPFC voxel, there were no differences in baseline metabolite levels. Thus, our study suggests that excitatory (E) glutamate response mechanisms to CBD are comparable regardless of diagnosis; whereas inhibitory (I) GABA response pathways are altered in ASD.

### Effect of CBD on Glx

The region including and surrounding the BG is richly innervated by a web of excitatory pyramidal neurons alongside GABAergic inhibitory projection neurons and glia cells [33]. The increase in Glx triggered by CBD in both groups could therefore have resulted from CBD binding to neuronal TRPV1 receptors. Subsequent activation of pyramidal neurons [15] may potentially have contributed to the altered Glx metabolite levels in the BG captured by MRS. Cannabinoid activation of TRPV1 receptors on microglia could also theoretically upregulate microglial activity and migration, leading to extracellular vesicular shedding and augmentation of Glx levels [34]. However, this is speculative, given the rapid desensitisation of TRPV1 receptors after activation [35].

In the DMPFC, glutamatergic pyramidal neurons predominate, with relatively fewer GABAergic interneurons (ratio ~4.7:1) [17]. Here, CBD reduced Glx in each group. One possible explanation for this is that CBD suppressed the activity of prefrontal glutamatergic neurons via their 5-HT_1A_ receptors [17, 18], thereby reducing Glx levels. Preliminary evidence has linked impaired TRPV1 signalling to the ASD risk gene SHANK3, and 5-HT anomalies, including 5-HT_1a_ receptor dysfunction, to ASD [36]. Despite this, we found no group difference in Glx response to CBD. This implies that glutamate targets of CBD in the BG and DMPFC in idiopathic ASD are no different from those in neurotypicals.

### Effect of CBD on GABA+

In contrast, CBD increased GABA+ levels in the BG and DMPFC voxel in neurotypicals, but decreased GABA+ levels in the BG and (markedly so) in the DMPFC voxel of autistic adults. The causes of group differences in GABA+ response are unknown. However, it may be partially explained by ASD-related alterations in CBD targets. For example, the expression of the CBD interneuron GPR55 receptor is reduced in the cortex in the valproic rat model of ASD [37]. Another explanation could be more general disruption to GABA pathways in ASD. For instance, a reduction in the activity of the rate-limiting GABA synthesising enzyme glutamic acid decarboxylase (GAD) [38], and genetic anomalies in GABA receptors [39] have been reported in ASD. Since MRS GABA+ is thought to reflect metabolic (intracellular) and extracellular GABA+ levels, rather than GABAergic synaptic transmission [40, 41], further studies are required to back-translate our results into preclinical models to dissect exactly what underpins the atypical cortical and sub-cortical GABA+ response to CBD in ASD; and what is the impact on excitatory and inhibitory system activity. For example, Kaplan and colleagues have reported that CBD appears to restore GABAergic neurotransmission in an animal model of Dravet syndrome [14]. Despite the limitations of resolution using MRS, the present findings, together with our previous finding of atypical prefrontal GABA responsivity to the glutamate-GABA acting drug riluzole, clearly point to an alteration in the *dynamics* of GABA, but not glutamate, systems in ASD. This observation may not only have aetiological relevance, but also add to the evidence that the GABA system may be a tractable treatment target in ASD [42, 43].

### Cortico-striatal systems (in ASD)

The CBD-induced shift in cortical and subcortical Glx and GABA+ levels may influence excitation and inhibition, although MRS does not tell us directly about excitation or inhibition at the level of the synapse. Nevertheless, this shift in metabolites could potentially have widespread implications for brain function and behaviour. This is because the BG (and the thalamus and insula) and DMPFC form part of a circuit that is heavily dependent on glutamatergic excitation and GABAergic inhibition and supports and regulates a range of cognitive processes. In brief, in the neurotypical brain, the BG receive input from the (insular) cortex, brainstem, and thalamus. Cortical input is predominantly excitatory [44], but BG output nuclei act via a direct monosynaptic GABAergic and an indirect polysynaptic glutamatergic pathway [45]. Projection neurons from the output nuclei provide GABAergic tonic inhibition to thalamocortical and brainstem neurons to complete a ‘loop’ [45, 46]. In ASD, however, neuroimaging studies have revealed reduced WM ‘integrity’ especially in prefrontal tracts [47], and abnormal ‘functional integration’ of the BG and the DMPFC. This is thought to partly explain why multiple processes dependent upon cortico-striatal loop integrity, such as socio-emotional, motor, and reward processing, are altered in ASD [48, 49]. Our results suggest that the structural and functional differences previously reported in MRI studies of cortical-subcortical systems in ASD extend to atypical E-I response to pharmacological challenge.

### Implications

The corollary of our observations is that because CBD ‘shifts’ glutamate and GABA+, it may affect glutamatergic excitation and GABAergic inhibition, and thereby impact on brain function. We did not directly test this here, but some support for this proposition comes from a recent report that CBD increases prefrontostriatal functional connectivity in neurotypical controls [50]. However, our results predict that the direction of a functional response to CBD may be distinct in autistic individuals, and this warrants further investigation.

Our results reinforce the fact that we cannot expect the actions of a drug tested in a typically developing population to be replicated in people with neurodevelopmental conditions. For example, we have previously reported a link between disrupted functional connectivity and an atypical MRS GABA+ response to pharmacological E-I challenge through riluzole in ASD but not in controls [19]. However, unlike CBD, riluzole increased prefrontal GABA+ in ASD. Together with our current findings, this suggests that GABA+ can be shifted bi-directionally in cortical-subcortical systems in adults with ASD. This is encouraging, as we can now begin to build a repertoire of drugs that elicit a biological response in ASD. This tactic will be critical given the heterogeneity of the autism spectrum, where a ‘one-drug-fits-all’ approach is unlikely to succeed. Thus, our next steps will be to examine whether acute drug response allows us to (i) identify more pharmacologically homogeneous sub-groups within ASD; and (ii) predict clinical responsiveness to sustained treatment.

### Limitations

We acknowledge that our study has important limitations. First, here we measured MRS bulk amounts of Glx and GABA+ in the chosen voxels of interest. This did not allow us to reliably discern the specific contributions of different compounds (glutamate and glutamine) contributing to the Glx signal. Moreover, at 3T, we were limited to draw inferences about intra-cellular and extra-cellular metabolite levels from our findings. Future studies with higher resolutions and magnetic field strengths are required to address these questions.

Second, we only included adult male subjects with an IQ above 70, and with no epilepsy or comorbid psychiatric conditions. This step was taken to ensure the homogeneity of our study sample and to make sure that observed effects were related to ASD rather than a comorbidity of ASD. However, this also limits our ability to extend our findings to the general ASD population, which is characterised by heterogeneity and the presence of psychiatric and neurological comorbidities. Future studies should attempt to replicate our findings in larger and more diverse population samples, and especially include women.

Third, our participant sample was relatively small. This can be attributed to our strict recruitment criteria (e.g. no use of illicit substances in the month leading up to and during the study). It is also influenced by difficulties inherent in time-intensive repeated-measures studies involving drug administration, e.g. high drop-out rates. Finally, also contributing to the modest sample size were our rigorous data quality criteria, e.g. exclusion of scans based on head motion, known to be a difficulty in ASD. That said, our sample size was comparable (or bigger than) previous MRS studies in ASD [19, 21]. Moreover, each individual in our study had two scans and thus acted as their own ‘control’ to reduce inter-subject variability and to increase statistical power.

Fourth, in this study we only investigated the impact of acute CBD administration on brain. We cannot extrapolate from the effects of a single dose to the impact of repeated administrations, e.g. as a therapeutic option in ASD, for several reasons. For instance, chronic CBD administration may result in a steady state, wherein the brain system plasticity equilibrates to the presence of CBD. Future studies should thus investigate the impact of long-term treatment with CBD on brain and behaviour.

## Conclusions

In summary, we report that CBD can ‘shift’ levels of Glx and GABA+. These metabolites contribute to the regulation of excitatory and inhibitory neurotransmission in *both* the typical and the autistic brain. However, our study also demonstrated that the atypical (autistic) brain reacts differently to CBD challenge of GABA+. Our findings that the GABAergic system is distinct in ASD, but can be shifted, is relevant both to our understanding of causal mechanisms and to the discovery of treatment targets in ASD. Additional studies will be required to (i) identify the neural basis of the response to acute CBD challenge, including potential pharmacologically homogeneous sub-groups within the autistic spectrum; (ii) examine potential functional consequences of CBD challenge in terms of inhibition, brain network activity, cognition, and behaviour; and (iii) investigate whether an acute response to CBD could predict the effects of sustained treatment in ASD.

## Supplementary information

Consort Flow diagram
