# Supplementary material for: Effects of cannabidiol on brain excitation and inhibition systems; a randomised placebo-controlled single dose trial during magnetic resonance spectroscopy in adults with and without autism spectrum disorder
Source: Neuropsychopharmacology. 2019 Feb 6;44(8):1398–405. doi: 10.1038/s41386-019-0333-8 (PMC6784992; doi:10.1038/s41386-019-0333-8)
Supplement: Supplementary file 1 — Consort Flow diagram [file 41386_2019_333_MOESM1_ESM.doc]

**
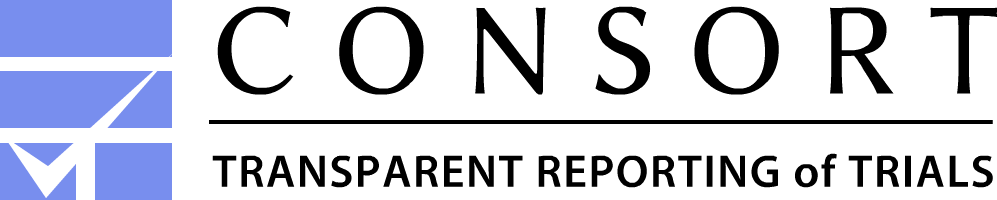
**

**CONSORT 2010 Flow Diagram**

**Allocation**

**Analysis**

**Follow-Up**

**Enrollment**

Assessed for eligibility (n=100)

Excluded (n=62)

♦  Not meeting inclusion criteria (n=62)

♦  Declined to participate (n=0)

♦  Other reasons (n= 0)

Analysed (n=23)
♦ Excluded from analysis (give reasons) (n=2) participants excluded due to positive result in drug screening

Lost to follow-up (give reasons) (n=0)

Discontinued intervention (give reasons) (n=1) participant unwilling to continue due to discomfort during MRI scan

Allocated to placebo before CBD (n=23)

♦ Received allocated order (n=23)

♦ Did not receive allocated intervention (give reasons) (n=0)

Lost to follow-up (give reasons) (n=0)

Discontinued intervention (give reasons) (n=0);

Allocated to CBD before placebo (n=16)

♦ Received allocated order (n=16)

♦ Did not receive allocated intervention (give reasons) (n=1); CBD not available

Analysed (n=16)
♦ Excluded from analysis (give reasons) (n=2) participants excluded due to positive result in drug screening

Randomized (n=38)
